# *Mycobacterium marinum* Infection after Iguana Bite in Costa Rica

**DOI:** 10.3201/eid2906.230062

**Published:** 2023-06

**Authors:** Jordan Mah, Kyle Walding, Brooke Liang, Laurence Rinsky, Roshni Mathew, Indre Budvytiene, Niaz Banaei

**Affiliations:** Stanford University School of Medicine, Stanford, California, USA (J. Mah, K. Walding, B. Liang, L. Rinsky, R. Mathew, N. Banaei);; Stanford Health Care, Stanford (J. Mah, I. Budvytiene, N. Banaei)

**Keywords:** *Mycobacterium marinum*, iguana bite, reptile bite, reptile zoonosis, nontuberculous mycobacteria infection, zoonoses, bacteria, Costa Rica, tuberculosis and other mycobacteria

## Abstract

Infections after reptile bites are uncommon, and microbial etiologies are not well defined. We describe a case of *Mycobacterium marinum* soft-tissue infection after an iguana bite in Costa Rica that was diagnosed through 16S rRNA sequencing and mycobacterial culture. This case informs providers of potential etiologies of infection after iguana bites.

Zoonotic infections associated with animal bite injuries are common and can result in severe illness (*1*,*2*). Approximately 5 million animal bites occur annually in North America, and 10 million injuries occur globally from dog bites alone (*2*,*3*). Pathogens causing infections after dog or cat bites are well described; pathogens from other animal bites are less well defined, although their oral microbiota are known (*1*). We report a case of cutaneous *Mycobacterium marinum* infection after an iguana bite to inform clinicians of potential infectious etiologies of lizard bites.

A previously healthy 3-year-old girl was on vacation with her family in Costa Rica. She was eating cake on the beach when an iguana approached her. While attempting to take the cake, the animal bit the dorsum of her left hand. She was immediately taken to a local clinic and found to have a single, superficial bite wound over the dorsum of her third metacarpal. The wound was immediately disinfected and irrigated; she was prescribed a 5-day course of oral amoxicillin. The family returned to the United States after the incident. Her wound completely resolved over the ensuing days without immediate complications.

Five months after the bite, her parents noted a small lump on the dorsum of her left hand that was not present previously. The child was otherwise well. The lump became progressively larger, erythematous, and mildly painful over the next 3 months (Figure, panel A). Because of persistent symptoms, her parents sought medical attention at Stanford Medicine Children’s Health (Stanford, California, USA). Although ultrasound demonstrated findings suggestive of a ganglion cyst (Figure, panel B), the location and symptoms were not consistent with this diagnosis. She saw an orthopedic surgeon who, given the progression and unusual clinical features, performed excision of the mass.

**Figure:**
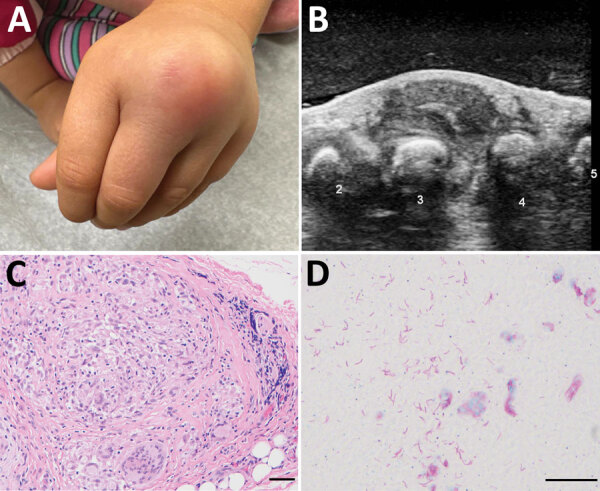
Gross and microscopic features of a mass involving the dorsum of the left hand in a 3-year-old girl with *Mycobacterium marinum* infection after iguana bite in Costa Rica. A) Erythematous lump over third and fourth digits. B) Ultrasound image, with numbers labeling the digits. C) Hematoxylin and eosin–stained soft tissue showing granulomatous inflammation. Scale bar indicates 10 µm. D) Fite stain highlighting numerous mycobacteria in an area of necrosis. Scale bar indicates 20 µm.

Surgical excision revealed a 2-cm, thick-walled mass adherent to the extensor tendons of the third and fourth digits, with extrusion of a thick, white, purulent material. Histopathology revealed extensive necrosis and necrotizing granulomatous inflammation with acid-fast bacilli seen by Fite staining (Figure, panels C, D). Bacterial 16S rRNA sequencing identified a sequence with 100% identity to *M. marinum* (GenBank accession no. OQ249694). Mycobacterial culture (Middlebrook 7H11 agar) incubated at 30°C grew photochromogenic colonies after 2 weeks that were consistent with *M. marinum*. The patient was started on rifampin and clarithromycin and gradually improved over the next 2 months.

Literature on the microbiologic etiologies of infected human wounds secondary to iguana bites is scarce; *Serratia marcescens* was reported in 3 cases and *Staphylococcus aureus* in 1 other (*1*). *Salmonella* *enterica* is a consideration for reptiles in general because 75%–90% of both wild and captive reptiles (including snakes, turtles, and iguanas) are colonized (*4*,*5*). Several studies have demonstrated that domestic reptiles can also harbor nontuberculous mycobacteria (NTM) because of their ubiquitous environmental presence (*4*). In a study of healthy pet reptiles, many were found to harbor NTM such as *M. fortuitum*, *M. peregrinum*, and *M. chelonae* (*6*). Reptiles can be asymptomatic carriers or can have NTM disease; cutaneous manifestations are the most common, with granulomatous lesions seen on histopathology (*4*,*6*). In this case, although the iguana is the most plausible source of *M. marinum*, we cannot rule out the possibility that the patient’s wound was inoculated from an environmental source.

*M. marinum*, a slow-growing photochromogenic NTM, is an established environmental pathogenic mycobacterium found in fresh water and salt water (*7*). *M. marinum* causes necrotizing granulomatous disease in humans, where its immunopathogenesis mimics that of *M. tuberculosis,* with which it shares considerable genetic homology (*7*). In humans, *M. marinum* is associated with occupational or recreational exposures after a skin injury where a contaminated water source enables direct inoculation (*7*). *M. marinum* is taken up by local macrophages and, like *M. tuberculosis*, uses the type VII secretion system ESX-1 (ESAT-6 secretion system 1), escaping the phagosome into cytoplasm and triggering an inflammatory response to spread to other macrophages (*8*). 

*M. marinum* causes disease in immunocompetent and immunosuppressed hosts; however, the incidence of cutaneous infections among children is low (*9*). The incubation period ranges from 3 weeks to 9 months, and symptoms are usually minimal and localized; systemic symptoms are generally absent (*7*). Common manifestations of *M. marinum* infections include subacute to chronic papulonodular skin lesions on the hand with a sporotrichoid spread, as the infection spreads along lymphatic vessels to regional lymph nodes (*7*). NTM, including *M. marinum,* are resistant to β-lactams because of β-lactamases, decreased cell permeability, and low affinity to penicillin-binding proteins, explaining why this patient did not respond to amoxicillin (*7*).

Isolation of *M. marinum* in culture is challenging because it is slow growing, requiring 28°C–32°C for optimal growth in vitro and incubation over several weeks (*7*,*10*). For this reason, bacterial sequencing is increasingly used for diagnosis because of its rapidity, sensitivity, and specificity. The cold-blooded nature of iguanas might enable them to serve as reservoirs for temperature-sensitive *M. marinum* (*4*). The genotypic and phenotypic evidence of *M. marinum* infection after an iguana bite in this report could inform clinicians of less commonly known bacterial etiologies after unusual zoonotic exposures.
